# Performance of the New 2019 European Alliance of Associations for Rheumatology/American College of Rheumatology Systemic Lupus Erythematosus Classification Criteria in a Large Unicentric Cohort

**DOI:** 10.3390/diagnostics12112778

**Published:** 2022-11-14

**Authors:** Ana Maria Gheorghiu, Cristina Vrancianu, Iuliana Conea, Aida Boca, Madalina Bolboceanu, Cristiana Draganescu, Mihai Bojinca, Ioan Ancuta

**Affiliations:** 1 Internal Medicine and Rheumatology Department, Cantacuzino Clinical Hospital, 020475 Bucharest, Romania; 2 Department 5, Carol Davila University of Medicine and Pharmacy, 020021 Bucharest, Romania; 3 Internal Medicine Department, Sf. Ioan Hospital, 042122 Bucharest, Romania

**Keywords:** systemic lupus erythematosus, classification criteria, performance, specificity, sensibility

## Abstract

Background: The recently published 2019 American College of Rheumatology/European Alliance of Associations for Rheumatology (ACR/EULAR) classification criteria for systemic lupus erythematosus (SLE) were developed to increase the reliability and identification of SLE, especially in early disease. With the emergence of several new drugs for SLE, identifying and treating patients early are more important than ever. Methods: Data of 446 SLE patients evaluated in our center between 1996–2019 and 226 controls with other autoimmune diseases evaluated between 2001–2022 were retrospectively analyzed. The sensitivity and specificity of the 2019 ACR/EULAR criteria were compared to the 2012 SLICC and the 1997 ACR criteria. Results: The 2019 ACR/EULAR criteria showed very good sensitivity (86.6%) compared to the 1997 ACR criteria (76.7%), *p* < 0.001, with a trend toward significance compared to the 2012 SLICC criteria (83.6%), *p* = 0.072. Their sensitivity remained high (87.6%) in patients with a short disease duration. The specificity of the 2019 ACR/EULAR criteria (91.2%) was statistically lower than the 2012 SLICC (96.0%) and 1997 ACR criteria (95.1%), *p* = 0.007 and *p* = 0.012, respectively, but still had a very high value. A total of 22 controls (9.7%) fulfilled at least one set of criteria (15 patients with MCTD, 5 with UCTD, and 2 with SSc). Conclusion: In this large real-life cohort, the 2019 ACR/EULAR criteria had very good performance compared to the 2012 SLICC and 1997 ACR criteria.

## 1. Introduction

Systemic lupus erythematosus (SLE) is a complex autoimmune disease of unknown etiology that can involve multiple organ and systems, such as the skin, joints, and kidneys as well as the cardiovascular, respiratory, hematological, and neurological systems [1]. The clinical and serological heterogeneity and the variability of the presentation make lupus well known for its ability to mimic other diseases [1]. Nonspecific clinical manifestations, especially in the early stages of the disease, lead to a late diagnosis that can potentially delay treatment and lead to poorer clinical outcomes [2]. While there are no diagnosis criteria for SLE, classification criteria exist and are needed to recruit patients with similar/comparable clinical and laboratory characteristics for research purposes and clinical trials [3], but they are also useful in clinical practice as guides for diagnosis. The first American College of Rheumatology (ACR) classification criteria for SLE were proposed in 1971 [4], but they were revised at least two times in 1982 and 1997 and later amended further [5,6,7]. The 1997 ACR criteria are based on 11 items; the presence of at least 4 of them serially or simultaneously, during any interval of observation, is required for the classification of SLE [7]. They were designed for research rather than clinical purposes and have several limitations [8]. In 2012, the Systemic Lupus International Collaborating Clinic (SLICC) classification criteria were developed based on real-life patient scenarios and included 17 variables [9]. They have been validated in another group of SLE patients and controls. They included more specific clinical features and also new immunologic tests proposed for use in routine evaluation: low levels of serum complement components, the presence of anti-beta-2 glycoprotein I antibodies, and the direct Coombs test [9,10]. The SLICC-12 criteria are fulfilled by either of the following: (a) biopsy-proven lupus nephritis in the presence of antinuclear antibodies (ANA) or anti-DNA antibodies; or (b) satisfying 4 out of 17 criteria, including at least 1 clinical and 1 immunological criterion [9,11]. The SLICC criteria highlighted that SLE is, above all, an autoimmune disease, requiring at least one immunological criterion. However, these current classification criteria for SLE have better performance in patients with long-standing disease, which emphasizes the need for criteria that can identify the early onset of SLE [10,12]. Additionally, organ-dominant forms of SLE might also impose classification challenges [13]. The recently published 2019 European Alliance of Associations for Rheumatology/American College Of Rheumatology (EULAR/ACR) classification criteria for SLE acknowledged the importance of SLE immunological features and hypothesized that the presence of antinuclear antibodies (ANA) would be more helpful as an entry criterion than as a classification one [10]. If the required entry criterion (a positive level of antinuclear antibodies (ANA) at a serum dilution of ≥ 1:80) is met, seven weighted clinical (constitutional, mucocutaneous, arthritis, neurologic, serositis, hematological, and renal) and three weighted immunological (antiphospholipid, complement, and highly specific antibody) domains follow. An accumulated score of 10 is considered a cut-off for a SLE classification [14]. One attribution rule, stating that an item should only be counted if there were no other explanations more likely than SLE, replaced exclusion criteria. Currently, more studies are needed in order to assess how these criteria perform in real-life scenarios.

The last few years have seen the emergence of several new drugs with promising results in randomized clinical trials, such as voclosporin, obinutuzumab, and last but not least, anifrolumab, which has recently been approved by the FDA for the treatment of active SLE. As such, identifying patients early in the course of the disease and ultimately starting treatment early is more important than ever. This is especially paramount in SLE patients with major organ involvement, when delaying specific therapy can result in serious irreversible damage.

Objectives: To compare the sensitivity and specificity of the new 2019 EULAR/ACR classification criteria for SLE with the 1997 ACR and the 2012 SLICC classification criteria in a large monocentric cohort of patients with SLE, diagnosed according to expert opinion.

## 2. Materials and Methods

All patients diagnosed with SLE who were evaluated in our department between 1 January 1996 and 31 December 2019 were included. A control cohort of patients with other autoimmune rheumatological diseases that may have features overlapping with SLE, evaluated between 1 January 2001 and 15 March 2022 in our department, was also included.

Medical charts were retrospectively reviewed using the hospital electronic database. For each patient, data regarding demographics, SLE manifestations up to diagnosis or the first visit in our clinic, date of diagnosis, and dates of first and last visits in our clinic were collected. Disease duration was defined as the duration of SLE manifestations up to the first visit in our clinic. All patients in the SLE and the control cohort gave written informed consent upon admission to our clinic, which included enabling the center to use their de-identified medical data in research projects; this study was approved by the local ethics committee.

Each of the three classification criteria as well as the control cohort were applied to the SLE. The definition of early disease used in our cohort was a disease duration of ≤3 years, similar to that of the validation cohort. In addition, as ours was a real-life cohort, the performance of the three classification criteria sets was also tested in patients with a shorter disease duration of ≤2 years. Sensitivity with 95% confidence intervals (95% CIs) of each criteria set was calculated against physician diagnosis. Specificity with 95% CIs was determined against patients with other autoimmune rheumatological diseases. The sensitivity and specificity of the 2019 EULAR/ACR classification criteria were compared to the 1997 ACR and 2012 SLICC classification criteria using the McNemar test for correlated proportions. A *p*-value of <0.05 was considered statistically significant. All statistical analyses were performed in the IBM SPSS Statistics v. 24.

## 3. Results

Characteristics of the patients with SLE are presented in Table 1. In short, 446 patients with SLE (413 women, mean ± SD age at diagnosis 35.9 ± 12.8 years) and 226 controls (192 women, mean ± SD age at diagnosis 44.0 ± 15.4 years) with systemic sclerosis (SSc) (29 patients, 12.8%), mixed connective tissue disease (MCTD) (18 patients, 8.0%), undifferentiated CTD (UCTD) (15 patients, 6.6%), rheumatoid arthritis (RA) (102 patients, 45.1%), SSc-RA overlap (2 patients, 3.0%), ankylosing spondylitis (54 patients, 23.9%), psoriatic arthritic (5 patients, 2.2%), and dermatomyositis (DM) (1 patient, 0.4%) were included. As expected, the number of younger female patients was higher in the lupus cohort compared to the control group. The most frequent SLE manifestations were mucocutaneous (415 patients, 93.1%), articular (349 patients, 78.3%), and hematological (338 patients, 75.8%), while the most seldom were severe neurologic (19 patients, 4.3%). There were significantly more patients with SLE who had constitutional symptoms, such as fever, renal, or neurological manifestations, compared to the controls. In the control group, the most frequent manifestations were articular (76.5%), which was comparable to the lupus cohort, partly due to the high proportion of RA patients. In addition, there were 15 patients with positive anti-dsDNA and anti-Sm antibodies, all with MCTD or UCTD; the patients with MCTD also had positive anti-RNP antibodies.

**Figure 1:**
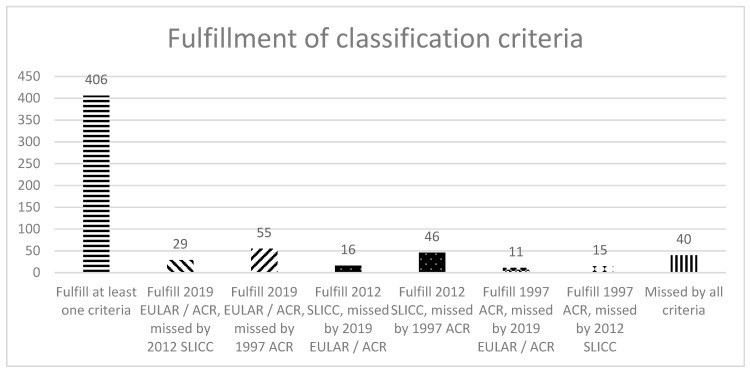
Number of SLE patients who fulfill or are missed by classification criteria. EULAR = European League Against Rheumatism; ACR = American College of Rheumatology; SLICC = Systemic Lupus International Collaborating Clinics.

Among the patients with SLE, 386 patients fulfilled the 2019 EULAR/ACR classification criteria, while 373 patients fulfilled the 2012 SLICC and 342 fulfilled the 1997 ACR criteria. The 2019 EULAR/ACR classification criteria had the best sensitivity of all criteria sets, showing a very good sensitivity of 86.6% (95% CI 82.9–89.5%), with a trend for significance compared to the 2012 SLICC criteria at 83.6% (95% CI 79.8–86.9%), *p* = 0.072, while the 1997 ACR criteria had a significantly lower sensitivity than both other criteria of 76.7% (72.4–80.5%), *p* < 0.001 for both comparisons, by a McNemar test. A total of 406 patients (91.0%) fulfilled at least one set of criteria (Figure 1); of the 40 patients who did not fulfill any classification criteria, 22 had a short disease duration of ≤ 3 years. Further details of how many patients were misclassified by each criteria set are given in Figure 1. If only ANA-positive patients (422 patients) are considered, the proportion of patients fulfilling the 2019 EULAR/ACR criteria increased to 91.5%, the 2012 SLICC criteria to 87.0%, and the 1997 ACR criteria to 78.9%.

There were 259 patients with SLE (58.1%) with a short disease duration of ≤ 3 years; of them, 247 patients had positive ANA assays. Among these patients with a disease duration of ≤ 3 years, 237 patients (91.5%) fulfilled at least one set of classification criteria. The sensitivity of the 2019 EULAR/ACR classification criteria in patients with a short disease duration was 87.6% (95% CI 82.9–91.3%), statistically similar to the 2012 SLICC criteria of 85.3% (95% CI 80.3.5–89.3%), *p* = 0.307, but slightly higher numerically, while the 1997 ACR criteria had a significantly lower sensitivity than both the 2019 EULAR/ACR and the 2012 SLICC criteria of 78.0% (72.4–82.8%), *p* < 0.000 and *p* = 0.001, respectively, by the McNemar test. If only ANA-positive patients with a short disease duration of ≤ 3 years were included, 94.7% of them would fulfill at least one set of classification criteria, with the 2019 EULAR/ACR criteria correctly classifying 91.9% of patients.

Moreover, since the aim in clinical practice was to classify patients as early as possible, 230 patients with SLE (51.6%) with a disease duration of ≤2 years were identified in our cohort; 220 of them had positive ANA. The sensitivity of the 2019 EULAR/ACR classification criteria remained high in these patients at 87%, increasing to 90.9% in ANA positive patients, versus 85.2% for the SLICC criteria, statistically similar (*p* = 0.523 by the McNemar test) but still numerically higher. Both classification criteria had better sensitivity than the 1997 ACR criteria at 76.5% and 79.1%, respectively, for ANA-positive patients (*p* = 0.000 for both).

In the control group, there were 20 patients fulfilling the 2019 EULAR/ACR classification criteria, and 9 and 11 patients fulfilled the 2012 SLICC and the 1997 ACR criteria, respectively. A total of 22 patients (9.7%) fulfilled at least one set of criteria; of them, 15 patients had a diagnosis of MCTD, 5 of UCTD, and 2 of SSc. The specificity (95% CI) of the 2019 EULAR/ACR was statistically lower than the specificity of the 2012 SLICC at 91.2% (86.7–94.5%) vs. 96.0% (92.6–98.2%), *p* = 0.007, and of the 1997 ACR criteria at 95.1% (91.5–97.6%), *p* = 0.012, but still had a very high absolute value. There was no difference between the 2012 SLICC and the 1997 ACR criteria, *p* = 0.625, by the McNemar test.

## 4. Discussion

The heterogenous manifestations of SLE often make its diagnosis challenging. In the early stages of the disease, it is all the more difficult to diagnose it as the symptoms can be similar to those of other conditions [15]. Currently, there are no diagnostic criteria for SLE, and the classification criteria generally have low sensitivity and high specificity, which means that the patients at the onset of the disease are not always identified. Therefore, it is important that the diagnosis be made as early as possible, given the life-threatening potential of the disease. The recent 2019 EULAR/ACR classification criteria have been proposed to improve the classification of SLE, especially in early disease, by preserving an increased sensitivity, similar to the SLICC 2012 criteria, while maintaining an increased specificity as is the case of the 1997 ACR classification criteria. The cohort used to validate the ACR/EULAR 2019 classification criteria included 1270 patients with SLE, comprising data from 21 SLE expert centers from 16 countries [10]. In our real-life clinical setting retrospective study, we identified a very good sensitivity of 86.6% of the new 2019 EULAR/ACR classification criteria, with a trend for significance in favor of the new 2019 EULAR/ACR classification criteria compared to the 2012 SLICC criteria and significantly higher compared to the 1997 ACR criteria. In the validation cohort, the sensitivity was 96.1% and the specificity was 93.4%, compared to 82.8% sensitivity and 93.4% specificity of the ACR 1997 and 96.7% sensitivity and 83.7% specificity of the SLICC 2012 criteria [10]. The slightly lower sensitivity in our SLE cohort compared to the validation criteria cohort may be due to the retrospective and observational nature of our study. This point is underlined by the fact that the sensitivity values for the new 2019 EULAR/ACR classification criteria found in our cohort are in line with other published data from observational studies [13]. In addition, other authors found a similar sensitivity of the new 2019 EULAR/ACR and the 2012 SLICC criteria when comparing them [14].

For patients with early disease (≤3 years disease duration) from the validation cohort, the 2019 EULAR/ACR classification criteria had better sensitivity than the 1997 ACR criteria: 97% compared to a significantly lower 81% [16]. The validation of the 2019 ACR/EULAR classification criteria for early disease is of significant importance because it ensures prompt inclusion of SLE patients in observational studies as well as in clinical trials. This, in turn, may prevent serious complications of the disease and irreversible damage, leading to a better prognosis. In our cohort, the sensitivity of the 2019 EULAR/ACR classification criteria in patients with a short disease duration was 87.6%, compared to a sensitivity of 85.3% for the 2012 SLICC criteria and 78.0% for the 1997 ACR criteria. While there was a significant statistical difference in the sensitivity of the new 2019 EULAR/ACR criteria and the 1997 ACR criteria for early onset cases in our cohort, we found only a numerical, but not statistically significant, difference when comparing them to the 2012 SLICC classification criteria. When analyzing a cohort of 690 SLE patients, Adamichou et al. [13] similarly found a significantly increased sensitivity of the 2019 EULAR/ACR (87.3%) and 2012 SLICC (91.4%) as compared with the 1997 ACR criteria (79.9%, *p* < 0.01 and *p* < 0.001, respectively) for patients with a disease duration of <3 years [13]. When we tested the performance of the three sets of criteria in patients with a disease duration of ≤2 years, we still found similarly good performance of the 2019 EULAR/ACR and SLICC criteria, suggesting these sets of criteria could be useful in clinical practice for identifying even earlier patients with SLE.

Another challenge that has long been recognized is that of the organ-dominant/limited forms of SLE, particularly consisting of neurological, nephrological, and hematological manifestations before sufficient criteria amass to meet classification criteria of the disease. These aspects were first addressed in the 2012 SLICC criteria through the introduction of the renal stand-alone criterion for recognizing renal-dominant lupus and is further acknowledged by the introduction of higher-weighted items in the 2019 EULAR/ACR ones, with the aim of reducing overrepresentation of some organs/domains, giving the new classification criteria the possibility to attain higher specificity [13]. However, although our results yielded an overall very high value at 91.2%, comparable to that found in other cohorts [13,14], the 2019 EULAR/ACR criteria showed statistically lower specificity compared to the 2012 SLICC (*p* = 0.007%) and the 1997 ACR criteria (*p* = 0.012%). Among the control patients with false positive results, most were patients with autoimmune rheumatic diseases, such as 20 SLE-mimicking MCTD and UCTD patients. The lower specificity found in our cohort could be due precisely to a higher weight in the 2019 EULAR/ACR criteria of domains, such as arthritis (6/10 points) or the hematologic domain (3–4/10 points), which are common manifestations of autoimmune rheumatic diseases, compared to the weight they had in the 2012 SLICC criteria. For example, a patient with positive ANA antibodies, who presents with arthritis and thrombocytopenia, would fulfill the 2019 EULAR/ACR classification criteria but not the 2012 SLICC criteria. Similar results were reported by Radin et al. [17], who retrospectively applied the 2019 SLE classification criteria to 133 female patients with UCTD and found that 22 patients (17%) fulfilled them. Early identification of SLE in patients with a previous diagnosis of UCTD might be important not only from a scientific point of view, but it could also influence the management of these patients, leading to closer follow-up, access to specific treatments (e.g., belimumab, rituximab, or anifrolumab), or eligibility to enter clinical trials [17]. It also underlines the need for tailored classification criteria or management guidelines for UCTD, which do not exist hitherto [17]. In addition, when retrospectively applying the 2019 SLE classification criteria to 214 antineutrophil cytoplasmic antibody (ANCA)-associated vasculitis (AAV) patients, Kwon et al. [18] found that 51 of them had positive ANA and 10 patients fulfilled the SLE criteria, the key indicator for differentiating AAV from SLE with histological confirmation.

In our clinical setting, the 2019 EULAR/ACR criteria performed best overall, with the best sensitivity-specificity balance (86.6%–91.2%) and an additional increase in sensitivity to 91.5% for ANA-positive patients, while also identifying early disease better than the previous sets of criteria. However, while they aid in clinical practice, classification criteria are especially developed for research purposes. It might not be possible to achieve the highest sensitivity combined with the highest specificity values simultaneously using only one set of criteria; it might be necessary to use a combination of criteria sets depending on whether sensitivity or specificity is of the highest importance [14].

There are several strong points of our study, such as the large SLE cohort of a specialized academic center, analyzed in a real-life clinical setting.

There are also disadvantages, such as the lack of recorded ANA assays for some patients and the possibility that not all the manifestations listed in the 2019 EULAR/ACR criteria were recorded, but these are inherent disadvantages of real-life data. In addition, the date at which each criteria manifestation had occurred was based on the information available in our clinical setting, so it was not possible in our cohort to calculate the date of the fulfillment of each set of criteria. Moreover, the retrospective nature of the study could potentially influence the reproducibility of the results. Finally, our control cohort contained lupus-mimicking patients with rheumatic systemic autoimmune diseases evaluated in our clinic (including MCTD), reflecting a real-life scenario, but this could represent a challenge for the specificity of any classification criteria, especially ones focused on increased sensitivity, such as the new 2019 EULAR/ACR criteria.

In conclusion, in our cohort, the new 2019 EULAR/ACR classification criteria had a very good sensitivity, achieving the goal of classifying more SLE patients with a shorter disease duration than previous ACR criteria. However, they had slightly lower specificity than previous criteria, especially for SLE-mimicking diseases such as MCTD. Classification criteria are frequently used in order to help diagnose patients with SLE. A combination of these criteria or a combination of their domains could lead to increased sensitivity, diagnosis of the patients earlier in the course of the disease, and even the development of diagnostic criteria. Future studies, ideally longitudinal, are needed to confirm these results.

## Figures and Tables

**Table 1:** Demographic and clinical characteristics of patients at diagnosis or first visit to our clinic.

Variables, *n* (%)	SLE (*n* = 446)	Controls (*n* = 226)	*p*-Value (*p* < 0.05)
Sex-female	413 (92.6)	192 (85.0)	0.02
Age at diagnosis, years, mean (SD)	35.9 (12.8)	44.0 (15.4)	0.00
Disease duration at first visit, years, mean (SD)	4.3 (7.5)	4.7 (9.7)	0.58
Fever	75 (16.8)	6 (2.7)	0.00
Renal involvement	112 (25.1)	2 (0.9)	0.00
Neurological involvement	19 (4.3)	0	0.00
Articular involvement	349 (78.3)	173 (76.5)	0.13
Mucocutaneous involvement	415 (93.0)	15 (6.6)	0.00
Serositis	113 (25.3)	7 (3.1)	0.00
Hematologic involvement	338 (75.8)	18 (8.0)	0.00
Autoantibodies			
Antinuclear antibodies	422 (94.6)	78 (34.5)	0.00
Anti-dsDNA	242 (54.3)	10 (4.7)	0.00
Anti-Sm	37 (8.3)	5 (2.3)	0.003
Antiphospholipid antibodies	74 (16.6)	3 (1.3)	0.00
Low complement	244 (54.7)	18 (8.1)	0.00

SLE = systemic lupus erythematosus; SD = standard deviation; Anti-dsDNA = anti-double stranded DNA; Anti-Sm = anti-Smith antibodies.

## Data Availability

The dataset used and/or analyzed during the current study is available from the corresponding author on reasonable request.
